# Saliva as a Diagnostic Tool for Systemic Diseases—A Narrative Review

**DOI:** 10.3390/medicina61020243

**Published:** 2025-01-30

**Authors:** Amelia Surdu, Liliana Georgeta Foia, Ionut Luchian, Daniela Trifan, Monica Silvia Tatarciuc, Monica Mihaela Scutariu, Corina Ciupilan, Dana Gabriela Budala

**Affiliations:** 1 Department of Oral Diagnosis, Faculty of Dental Medicine, “Grigore T. Popa” University of Medicine and Pharmacy, 700115 Iasi, Romania; 2 Department of Biochemistry, Faculty of Dental Medicine, “Grigore T. Popa” University of Medicine and Pharmacy, 16 Universitătii Street, 700115 Iasi, Romania; 3 St. Spiridon Emergency County Hospital, 700111 Iasi, Romania; 4 Department of Periodontology, Faculty of Dental Medicine, “Grigore T. Popa” University of Medicine and Pharmacy, 700115 Iasi, Romania; 5 Department of Dental Technology, Faculty of Dental Medicine, “Grigore T. Popa” University of Medicine and Phamacy, 700115 Iasi, Romania; 6 Department of Orthodontics, Faculty of Dental Medicine, “Nicolae Testemitanu” University of Medicine and Phamacy, MD-2004 Chisinau, Moldova; 7 Department of Morpho-Functional Science, Faculty of Medicine, “Grigore T. Popa” University of Medicine and Phamacy, 700115 Iasi, Romania; 8 Department of Dentures, Faculty of Dental Medicine, “Grigore T. Popa” University of Medicine and Phamacy, 700115 Iasi, Romania

**Keywords:** saliva, oral diagnosis, systemic disease, proteomics, genomics, periodontitis

## Abstract

Saliva has emerged as a powerful diagnostic tool due to its non-invasive collection, straightforward storage, and ability to mirror systemic health. This narrative review explores the diagnostic potential of salivary biomarkers in detecting systemic diseases, supported by examples such as salivary proteomics’ role in monitoring endocrine disorders, cancer, and viral infections. Advances in technologies like microfluidics, biosensors, and next-generation sequencing have enhanced the sensitivity and specificity of salivary diagnostics, making it a viable alternative to blood-based diagnostics. The review also evaluates challenges such as the need for standardized collection protocols, variability in salivary composition, and the integration of these technologies into clinical workflows. The findings emphasize the transformative potential of saliva in personalized medicine, especially for early disease detection and real-time health monitoring. Practical applications include its use in mass screenings and public health crises, highlighting saliva as a cornerstone for future advancements in non-invasive diagnostics.

## 1. Introduction

Hundreds of tiny, minor submucosal glands, as well as paired parotid, submandibular, and sublingual large glands, work together to create saliva, a complex fluid. Because it is non-invasive, easy to use, and less expensive than conventional diagnostic methods like blood and tissue samples, saliva is quickly becoming a popular choice for medical professionals [1]. Proteins, enzymes, hormones, nucleic acids (DNA, RNA, miRNA), and metabolites are only a few of the indicators found in its distinctive makeup that reflect the overall health or illness condition of the body. Saliva collection is a patient-friendly alternative to invasive medical procedures since it is simple and does not require specialized medical skills [2,3].

Salivary component alterations are a common symptom of systemic illnesses including cardiovascular problems, endocrine abnormalities, viral infections, malignancies, and neurological issues [4,5,6]. Because of these features, saliva can be used to monitor diseases and diagnose diseases early [7,8].

An exhaustive synopsis of the diagnostic capability of saliva for systemic disorders is the goal of this narrative review. It delves into the technological developments propelling salivary biomarkers ahead, their uses across many disease domains, and their biological foundation. Also, we discussed the barriers and restrictions that need to be overcome before salivary diagnostics may be used in clinical settings on a large scale. In order to improve healthcare outcomes and completely restructure diagnostics, this study fills in the gaps in our present understanding of saliva and its revolutionary potential.

This review included studies examining the diagnostic utility, clinical applications, or physiological properties of saliva. Eligible studies were original research articles, systematic reviews, and meta-analyses focused on salivary biomarkers, published in peer-reviewed journals within the past 20 years (2004–2024) to ensure relevance to current advancements. Studies were retrieved using PubMed, Scopus, and Web of Science databases as primary sources for literature search due to its extensive coverage of biomedical studies. Keywords such as “salivary diagnostics”, “saliva biomarkers” and “systemic disease detection” were employed in the search strategy. Studies were excluded if they did not focus on the diagnostic potential or clinical applications of saliva, were not published in peer-reviewed journals (e.g., conference abstracts, editorials, or commentaries), lacked clear methodologies or accessible data, were published in languages other than English, or were exclusively based on animal models without relevance to human applications.

## 2. Salivary Composition and Secretion

Saliva is 99% water and is an exocrine solution. Proteins make up 0.3% of the total, inorganic substances account for 0.2%, and organic molecules make up the remaining. Enzymes such as amylases, peroxidase, lipase, lysozyme, lactoferrins, kallikreins, cystatins, hormones, and growth factors are examples of organic components, whereas sodium, potassium, calcium, magnesium, chloride, and carbonates are examples of inorganic components [9]. Of the approximately 3000 protein components found in saliva, over 90% belong to the following classes: α-amylases, proline-rich proteins (PRPs; classified as acidic, basic, or basic glycosylated), mucins, salivary cystatins (also known as “S-type”), histatins, statherin, and P-B peptide. Roughly 200 proteins and peptides are composed of all these components and their derivatives [10].

The three pairs of main salivary glands—the submandibular (about 65%), parotid (about 20%), and sublingual (about 5–7%)—secrete secretions that are the primary source of saliva (about 90%). Between 0.5 and 1.5 l of saliva is produced everyday by a healthy adult [11].

Olfactory sensations, chewing, and oral mechanoreception all work together to stimulate saliva production. Afferent sensory nerves transmit nerve-mediated impulses to the central nervous system, whereas parasympathetic and sympathetic autonomic nerves convey efferent signals to the salivary glands [12,13,14,15,16]. Both the sympathetic and parasympathetic neural systems can stimulate salivary gland production, although the latter has stronger and more long-lasting effects [17,18,19,20].

Sympathetic activation increases the flow of volume and amylase by increasing the stimulation of alpha receptors by norepinephrine, which in turn causes smooth muscle contractions. The effects of norepinephrine, which triggers the cyclic adenosine monophosphate cascade and works on beta receptors, include an increase in protein kinase A (PKA) activity, amylase synthesis, and transient saliva volume flow [21,22,23,24,25].

## 3. Salivary Secretion Disorders

On a daily basis, an adult produces 1/2 L to 2 L of saliva, with just a tenth of that quantity coming from production throughout the night [26].

Saliva production can be affected by a wide range of medical conditions, therapies, and drugs. This can happen for a variety of reasons, some of which include aberrant salivary components, reduced salivary flow, or the perception of dry mouth (xerostomia) despite normal salivary flow or sialorrhea. In view of the numerous functions played by saliva, any disorders of its secretion are reflected by an array of pathological changes, not only in the oral cavity but also within the alimentary tract [27,28,29,30].

➢ *Xerostomia*

The vast majority of xerogenic drug-related occurrences of dry mouth occur as a result of medication usage [31]. Medications for a wide range of conditions, both mental and physical, are available here. Some of these conditions include anxiety, depression, nausea, hypertension, heart disease, bronchodilators, and muscle relaxation [32]. The use of psychiatric pharmaceuticals is the biggest explanatory factor for all patients, whereas the primary reasons for xerostomia in elderly hospitalized patients and elderly outpatients with respiratory disorders are cardiovascular medications [33].

The synergistic effects of medication combinations and the higher incidence of chronic illnesses needing pharmacological therapy in older adults are two of the many reasons that lead to xerostomia, even if saliva flow does not always diminish with age [34,35,36]. The underlying medical conditions that need the administration of these medications might often have long-lasting effects, even though drug-induced xerostomia is usually reversible [37,38,39].

➢ *Sialorrhea*

The most common causes of sialorrhea are problems with neuromuscular control, excessive production of fluids, impaired sensory function, or impaired anatomical (motor) control. Additional potential reasons of sialorrhea include malignancies of the head and neck, surgical or radiation-induced abnormalities in the head and neck or the upper gastrointestinal tract, and other similar conditions. In addition, sialorrhea can occur in patients undergoing palliative care, as well as those who are ventilated and/or have had a tracheotomy. Some medications, such as pyridostigmine and other parasympathomimetic, as well as neuroleptics, particularly clozapine, can cause drug-induced sialorrhea.

Collaborative efforts are optimal for treating sialorrhea. When seeing a patient for primary care, doctors often take a thorough medical history and perform a physical exam, paying close attention to how excessive salivation affects the patient’s quality of life and whether or not there is a way to make it better [40,41,42].

## 4. Role of Saliva

### 4.1. Immunological Functions

Saliva shields oral tissues from harmful substances via its many physical, physicochemical, and chemical agents. There are always immune and non-immune components in the mouth, but it discards both types of microbes and their byproducts into the digestive tract [43].

Saliva works as a defense mechanism due to its quantities of thiocyanates, myeloperoxidase, lactoferrin, salivary peroxidase, and lysozyme [44]. Clinical applications of salivary secretions take advantage of their naturally protective properties, such as (1) the development of diagnostic reagents and tests for local and systemic diseases, (2) the formulation of artificial saliva for the management of salivary dysfunction, and (3) the development of topical vaccines for oral diseases [45]. The functions inside the epithelial barrier of mucosal defense are significantly impacted by salivary mucins, which are known to be essential for the preservation of oral cavity health [46].

### 4.2. Lubricant Functions

An efficient mechanism for lubricating and preserving both hard and soft tissues is provided by the complex mixture of components found in saliva [46,47]. While at repose, saliva mostly performs its lubricating and antibacterial duties, which include flushing the teeth and removing harmful substances from the mouth [48,49,50].

Active transportation, passive diffusion, and ultrafiltration are the pathways by which proteins can enter the salivary glands from the blood circulation. Some of these proteins are subsequently released into saliva, which has the ability to act as a biomarker for many disorders [51]. An important part of establishing the oral microbiota is the conditioning film that saliva applies to the hard and soft tissues of the mouth. This film determines the first adhesion of microbes [52,53].

### 4.3. Taste and Digestive Functions

The only way for compounds with a flavor to interact with taste receptors on taste buds is for them to be in a solution. A crucial function of saliva is to transport the tastant from the mouth to the taste receptors. It is necessary for the tastant to diffuse through the salivary film that typically covers the oral mucosa once it approaches a taste bud [54].

Elevated taste thresholds can be caused by damage to the taste buds that occurs when salivary flow is severely restricted, as in disorders like Sjogren’s syndrome, and the oral mucosa is very dry [55]. Additionally, due to the destruction of the taste and smell receptors that is often linked with chemotherapy and head and neck radiation therapy, patients often have difficult-to-treat changes in taste [56]. Therefore, one of the primary roles of saliva is to dilute and expel food acids and sugars, oral germs, and other potentially harmful substances from the mouth. This process is also known as salivary or oral clearing [57,58]. On average, there is 1.1 mL of saliva in the mouth before swallowing, and around 0.8 mL of saliva remains after swallowing [59,60,61].

Containing mucins, enzymes, antibacterial proteins, and immunoglobulins, the remaining saliva forms a thin, movable coating that covers the oral surfaces and protects the oral cavity [62]. The bicarbonate, phosphate, and protein composition of saliva is the source of its buffer capacity, which allows it to neutralize acids. The saliva buffers dietary acids and acids produced by bacteria fermenting carbohydrates, keeping the salivary pH at a physiologically constant range of 6.5–7.4. This slows down the demineralization process in teeth [63].

### 4.4. Saliva Proteome Functions

**Figure 1:**
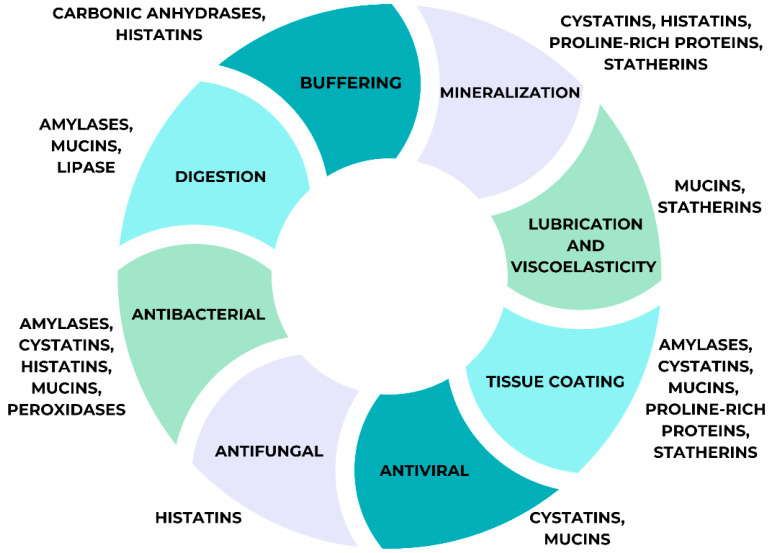
Saliva multifunctionality.

Whole saliva proteomes are more reactive than serum proteomes to a wide range of physiological and pharmacological treatments. To begin, the production of proteins in saliva is regulated by the nervous system, and the number of proteins released is influenced by external stimuli. The second step is the oral environment, where a variety of enzymes produced by both the host and bacteria alter the proteins coming out of the glandular ducts in saliva [64]. Glycosylation, phosphorylation, and proteolysis are steps in the post-translational processing that follow the transcription and translation of genes for salivary proteins in the glands. This results in salivary proteins that are passed down across families of individuals with very similar structures [64,65]. A summary of all salivary functions is represented in Figure 1 below.

## 5. Saliva as a Diagnostic Tool

In addition to all these crucial roles, saliva acts as a solvent for peptides, ions, and metabolites that are either secreted or formed during the breakdown of drugs and endogenous chemicals. These biomarkers can be used to diagnose diseases, and the molecular makeup of saliva reflects these states.

A saliva analysis has the potential to aid in the identification of endocrine disorders, cancers, autoimmune illnesses, infectious diseases, and genetic disorders; it may also be used to monitor medication usage and determine treatment dosages [65].

The therapeutic uses of salivary diagnostics have shown significant promise, and saliva is a promising body fluid for early identification of illnesses [66]. Improvements in detection technologies and combinations of biomolecules with clinical significance have the potential to make saliva the sample of choice for first-line diagnostics [67]. Along with clinical tests, monitoring salivary biomarkers for oral and systemic disorders could evolve into an essential component of epidemiological research, as synthetized in Table 1 below [68].

### 5.1. Oncology

Oncology is a branch of medicine that deals with the prevention, diagnosis, and treatment of cancer. Saliva serves as a useful diagnostic means in the early detection of various cancers such as oral cancer, pancreatic cancer, breast cancer, lung cancer, or gastric cancer.

Among all human malignancies, pancreatic cancer has the poorest five-year survival rate, affecting 44,030 people every year [69]. A study conducted by Humeau et al. investigated the possibility that salivary microRNAs (miRNAs) discriminate against patients with non-operable pancreatic tumors as compared to patients with precancerous lesions, inflammatory diseases, or healthy controls [70].

Saliva microbiota can differentiate between healthy people and those with pancreatic adenocarcinoma, according to research by Wei et al. [71]. There was an inverse relationship between the quantity of *Streptococcus* and *Leptotrichia* and the risk of pancreatic cancer. Protective factors for the detection of pancreatic adenocarcinoma were found to be Veillonella and Neisseria.

Early identification of pancreatic cancer may be possible with a combination of symptoms and microbiota examination, since pancreatic carcinoma symptoms are often ambiguous. When designing probiotic therapy regimens to lower pancreatic cancer risk, understanding the spread of bacterial flora is a crucial first step.

Worldwide, gastric cancer ranks second in cancer-related fatalities and fourth in occurrences of cancer overall [72]. The delayed diagnosis is the major reason for the high death rate, as early stomach tumors usually do not exhibit any specific symptoms or are asymptomatic [73]. Salivary proteins cystatin B (CSTB), triosephosphate isomerase (TPI1), and deleted malignant brain tumors 1 protein (DMBT1) have all been associated to gastric cancer. With a sensitivity of 85% and a specificity of 80%, these markers might be utilized to distinguish gastric cancer patients from control people (*p* < 0.05) [74].

Furthermore, when comparing breast cancer patients to controls, there may be a positive correlation between salivary levels of protein CA15-3 and lung resistance protein (LRP) [75,76].

Patients face significant rates of death and morbidity from head and neck squamous cell carcinoma (HNSCC), which is the sixth most frequent malignancy globally [77]. Tumors in this category can be found in many different places, including the mouth, nose, paranasal sinuses, salivary glands, throat, and larynx [78].

In all cases of oral cancer and in 47–70% of other malignancies, oral squamous cell carcinoma can be diagnosed when saliva tests reveal somatic mutations on the TP53, PIK3CA, CDKN2A, HRAS, NRAS, or HPV genes. Saliva samples collected from individuals who have had treatment can also reveal tumor DNA, even before the onset of recurrence symptoms [79].

Oral squamous cell carcinoma patients’ saliva had an overexpression of miR-31 and decreased levels of miR-125a and miR-200a expression patterns, according to two studies [80,81]. The miR-31 gene in saliva can be used for disease monitoring specifically. Oral cancer patients experience persistently high levels throughout the disease, in contrast to non-cancerous premalignant lesions and healthy controls. After tumors were removed, researchers found that salivary miR-31 levels were reduced [81]. The expression level of miR-139-5p returned to its native form after the primary tumor was surgically removed from oral squamous cell carcinoma samples, which was much lower than in controls [82].

Salivary indicators for oral cavity cancer and other locations include an increase in tumor antigen CA15-3 and antibodies for tumor protein markers c-erbB2, CA-125, and p53 [83]. Other markers that may point to oral squamous cell carcinoma include increased IL-10 and IL-13 levels and reduced IL-1 receptor antagonist (IL-1ra) levels [84]. Similarly, compared to healthy controls, individuals with laryngeal cancer were found to have saliva that overexpressed soluble CD44 both before and after surgery [85].

Oral squamous cell carcinoma can be indicated by increasing porphyrin levels [86] and reduced valine, leucine, isoleucine, and phenylalanine levels [87].

Oral microbiota was investigated by Furquim et al. [78] in relation to oral squamous cell carcinoma risk in patients vs. healthy controls. Gingival disease (*Prevotella*, *Streptococcus*, *Prophyromonas*, and *Dialister*), oral graft-versus-host disease (GVHD) (*Firmicutes*), and oral mucositis were identified as having distinct microbial characteristics [78].

The problems that arise with tissue biopsy are not a concern when using liquid biopsy. Biofluids with molecular signatures emitted from diseased tissues can be easily accessed for the purpose of screening and monitoring individuals’ health information. By examining cell-free DNA in the blood, liquid biopsies can test for the existence of malignant cells. Rapid and cost-effective illness detection and information collection on treatment targets are made possible with the use of liquid biopsy. The obvious benefit of cell-free DNA over tissue biopsy is the absence of risk associated with the non-invasive collection process, which allows for repeated, longitudinal molecular analysis of cancer in areas of the body that surgeons have a hard time reaching [88].

Hypermethylation of the cyclin-dependent kinase inhibitor 2A was the primary focus of an investigation into KRAS gene mutations in patients with colorectal cancer conducted by Lecomte et al. [89]. A two-year survival rate of 100% was demonstrated in patients with KRAS mutations or CDKN2A gene promoter hypermethylation who did not have any detectable cell-free DNA. Furthermore, research demonstrated the usefulness of cell-free DNA as a prognostic biomarker for detecting colorectal cancer recurrence in patients [89]. A relapse of illness occurred within one year following surgery for individuals with evidence of cell-free DNA, according to a research by Spindler et al. [90]. Metastatic colorectal cancer patients who had a high proportion of cell-free DNA and KRAS mutations had a poor prognosis, according to this study [90].

There is a plethora of clinical uses for cell-free DNA in cancer therapy, including molecular profiling and diagnosis, therapeutic response tracking, resistance and tumor heterogeneity monitoring, postsurgical residual disease identification, and early cancer detection. There are, however, a few restrictions with liquid biopsy, as well. For the cell-free DNA tests to be truly useful in the clinic, particularly for detecting residual illness, monitoring therapy efficacy, or detecting early-stage cancer, they require more clinical validation [91].

Until the disease has progressed enough, the signs of cancer are generally vague. Screening, early detection, diagnostics, staging, and prognostics for cancer must be addressed immediately with the development of non-invasive, extremely accurate, and quick techniques. Recent improvements in omics analysis have been invaluable to the discovery of biofluid-associated cancer biomarkers; these findings have the potential to pave the way for a more precise and trustworthy evaluation of molecular target levels. It is possible to successfully connect salivary diagnoses with clinical diagnosis and explore their potential as biomarkers for histological grading and clinical staging of cancer.

### 5.2. Cardiac Diseases

There is a high case-fatality ratio associated with acute myocardial infarction, a frequent cardiovascular event. In the patient’s care strategy, time is an important issue. An important application of salivary diagnostics in cardiology includes acute myocardial infarction risk assessment in insulin resistance patients [92,93]. Therefore, in patients with precordial discomfort of less than 4 h, salivary alpha-amylase was found to be a diagnostic factor for acute myocardial infarction [94]. Furthermore, it was shown that screening individuals with acute myocardial infarction using nano-biochips derived from salivary proteins (such as C-reactive protein, myoglobin, and myeloperoxidase) was efficient [95].

The study by Floriano et al. [95] found that C-reactive protein containing salivary biomarker panels showed a good diagnostic capability (*p* < 0.0001) and a greater diagnostic capacity (AUC = 0.96) for acute myocardial infarction when paired with ECG than ECG alone.

Patients with cardiovascular disease had also lower amounts of α-2-HS-glycoprotein in their saliva, suggesting that the peptidome might be a useful tool for early detection of cardiovascular disease [96].

### 5.3. Immune Mediated and Inflammatory Skin Diseases

Currently recognized as a multisystemic illness, psoriasis is a skin ailment characterized by persistent inflammation that is mediated by the immune system. There is a need for dependable biomarkers that can identify a pathological condition and/or a treatment response because several pieces of evidence have connected psoriasis to comorbidities, including cardiovascular ones [97,98]. Saliva is an intriguing and controllable biological fluid; thus, researchers have focused on it [99].

Studies in psoriatic patients have only looked at a small subset of the salivary proteome, which contains 2000 proteins, because researchers have only targeted a small number of biomarkers thus far. Several studies measured the amounts of acute-phase proteins in saliva, including sAA, haptoglobin, and C-reactive protein (CRP). Because the C-reactive protein (CRP) is recognized to have a predictive value for the progression of psoriasis and is connected with the inflammatory character of the illness, its statistically significant elevation in psoriatic individuals is not surprising [100]. Similarly, haptoglobin levels in saliva were found to be elevated, which might indicate a local mechanism that protects against psoriasis. Twenty patients with psoriasis had salivary alterations that were associated with a greater concentration rate of sAA than the controls; however, these changes were unrelated to the severity or duration of the illness, according to Soudan et al. [101].

Psoriatic patients had reduced concentrations of transferrin and neutrophil gelatinase-associated lipocalin in their saliva compared to periodontitis patients and healthy controls, according to a Danish research [102].

Saliva samples from psoriatic patients were tested for several cytokines. Ganzetti et al. showed that compared to controls, psoriatic patients had greater levels of salivary IL-1β [101]. Other cytokines, including transforming growth factor (TGF)-β1, IL-8, interferon (IFN)-γ, IL-17A, IL-4, IL-10, monocyte chemoattractant protein (MCP)-1, microphage inflammatory protein (MIP)-1a, and MIP-1b were studied by the same author. The levels of salivary IL-1β, TNF-α, TGF-β1, and MCP-1 were considerably greater in psoriatic patients compared to controls [103].

Certain elements of the salivary profile of psoriasis, including elevated levels of certain cytokines and acute-phase proteins, are clearly developing, indicating that saliva may already serve as a legitimate and safe instrument for monitoring inflammation. Nonetheless, the data require validation and elaboration through additional studies involving larger sample sizes and broader salivary profiling.

A few acquired bullous dermatoses that can affect the skin and/or mucous membranes include bullous pemphigoid (BP) and pemphigus vulgaris (PV). A histological evaluation by the direct immunofluorescence of skin and/or mucosal biopsies is the most reliable method for diagnosing the two diseases. Due to the strong correlation between serum and salivary protein levels, an ELISA analysis of desmoglein 1 and desmoglein 3, two transmembrane desmosome proteins, in saliva was found to be a very sensitive and specific assay in 2006 by Andreadis et al. [104]. This makes it an ideal tool for diagnostic purposes, disease activity monitoring, and early identification of pemphigus relapses.

Achromic patches caused by the death of melanocytes in the skin or hair, or in both, are characteristic of vitiligo, an inherited skin disorder whose cause is unclear [105]. Studies examining saliva composition in vitiligo patients are few in scientific literature. The secretion of blood group-specific chemicals in the saliva of 76 vitiligo patients and normal controls was studied by Sehgal et al., who found that the secretors were more widely distributed in the saliva of the vitiligo patients [85].

### 5.4. Infectious Diseases

The stomach mucous is a typical habitat for the Gram-negative bacteria *Helicobacter pylori*. Endoscopy and the urea breath test can identify it in the stomach [106]. Nevertheless, it is advised against using this test to diagnose oral *H. pylori* infection because the oral cavity is home to several *Streptococcus* and *Campylobacter*-like bacteria that might provide false positive findings due to their urease-positive capabilities [107]. The use of polymerase chain reaction (PCR) for the detection of *H. pylori* in saliva is more effective due to these false positives [108].

Screening for viral nucleic acids is the standard method for determining if oral fluid specimens contain live viruses. Since viruses in the sample are rendered inactive during the first viral nucleic acid separation technique, approaches based on viral nucleic acids are both more sensitive and less infectious. On the other hand, detecting viral nucleic acid by amplification can be costly, time-consuming, and equipment-specific; furthermore, it is not certain to indicate a current infection. In order to get a final diagnosis, it may be necessary to perform a cultivation confirmation test [109].

Researchers have shown that oral samples can be just as useful as blood or urine samples when looking for nucleic acids, antibodies, and pathogen-specific antigens. Although target molecule concentrations in saliva are typically lower, amplification methods make it possible to identify viral indicators [110]. Antibody analyses based on saliva may identify a wide range of viruses at the proteome level, including HIV-1, measles, rubella, vesicular stomatitis, mumps, and hepatitis A, B, and C. Saliva seems to be a more sensitive indicator of human cytomegalovirus (HHV-6) than blood, according to the study by Nefzi et al. [111]. Salivary antibody levels can also be used to detect infections caused by the Morbillivirus, Paramyxoviridae (mumps), or Togaviridiae (rubella) [112].

Since the COVID-19 pandemic, when saliva-based diagnostic tests were first put into practice, there has been a large amount of debate concerning the use of saliva as a diagnostic tool for seasonal respiratory viruses [113]. Researchers have recently shown that nasopharyngeal specimens and saliva are very reliable in detecting respiratory tract infection pathogens including adenovirus, rhinovirus, enterovirus, human metapneumovirus, parainfluenza virus, bocavirus, and human coronaviruses, relative to other screening tests.

The use of saliva in molecular tests to identify respiratory viruses has been revived, despite its rarity in this context due to the popular belief that saliva is less sensitive than other fluids. Using the reverse transcription polymerase chain reaction, several investigations found that saliva had a higher detection rate for respiratory viruses than nasopharyngeal specimens [114,115]. Similarly, Kim et al. [116] found that nasopharyngeal specimens and saliva both exhibited comparable detection rates when employing multiplex polymerase chain reaction.

Molecular testing on saliva has the potential to attain diagnostic sensitivity and specificity comparable to or higher than that of nasopharyngeal swabs, according to recent scientific research. This finding is especially pertinent in view of the COVID-19 pandemic. The absence of uniformity in collecting and processing saliva may, unfortunately, impact SARS-CoV-2 detection. The posterior oropharyngeal region and the mouth are two examples of the many possible sources of saliva, each of which offers a distinct set of characteristics. Better methods are required to guarantee reliability, and another potential issue with this technology for diagnostic purposes is the difficulty in collecting and processing saliva owing to its high viscosity. Therefore, additional investigations need to focus on improving clinical research, defining standards, and confirming the accuracy of salivary diagnostics in order to improve the diagnostic and surveillance abilities of salivary biomarkers.

### 5.5. Diabetes Mellitus

An imbalance in glucose metabolism, brought on by insulin resistance, inadequate insulin production, or both, is the fundamental cause of metabolic illness known as diabetes. Patients with type 2 diabetes mellitus may be able to assess their glycemic control by observing their salivary α-2-macroglobulin levels, since there was a positive link between these two variables and hemoglobin A1c [117]. Having said that, those who suffer from type 2 diabetes have lower concentrations of melatonin in their saliva. It follows that salivary melatonin may play a pivotal role as a biomarker for diabetes diagnosis and management, given its significance in the disease’s pathophysiology [118].

Diabetes patients had noticeably elevated glucose and α-hydroxybutyrate levels, as well as notable shifts in carbohydrate, lipid, and oxidative stress levels, according to Barnes et al. [119]. Consequently, the metabolites may have a role in diabetes diagnosis, treatment, and prognosis.

In another study, patients with diabetes were significantly correlated with salivary glucose concentrations and HbA1c. This implied that people with diabetes mellitus may use their saliva to track their blood glucose levels [120].

Furthermore, Aitken-Saavedra et al. [121] found that HbA1 had a strong association with total protein quantity and inversely associated with saliva pH in type 2 diabetes mellitus. Measuring the qualitative and quantitative changes in saliva might be an affordable and efficient technique for observing patients with diabetes, a widespread long-term condition with numerous related complications.

### 5.6. Endocrinological Disorders

The content of melatonin and unconjugated steroid hormones may be evaluated using saliva, which has replaced other bodily fluids such plasma, serum, and urine as a diagnostic instrument in endocrinology [122]. In addition to its usage in diagnosing hirsutism, menstruation problems, adrenal and testicular tumors, steroidogenesis disorders, and controlling the effects of antiandrogenic medication, saliva is used to determine levels of estradiol and testosterone [123].

Salivary diagnostics now enables the following: endocrine function monitoring with dynamic testing (Dexamethasone), medication concentration and metabolism control (hormone replacement therapy), and free fraction determination for several hormones. The merging of these methods has the potential to drastically cut down on the expenses associated with costly hormonal endocrine investigations.

### 5.7. Renal Disorders

In a study of individuals with chronic renal failure, Venkatapathy et al. examined the relationship between serum and salivary creatinine [124]. Serum and saliva creatinine levels were greater in individuals with illness, according to their findings [124]. It is now possible to keep track of renal functions and estimate the success rate of dialysis in patients with end-stage terminal renal disease by determining the amounts of salivary creatinine in saliva [124].

### 5.8. Psychiatric Disorders

Alteration in hormones like cortisol and alpha-amylase have been shown by various researchers to be associated with anxiety-related disorders. Saliva analysis, which often involves cortisol assays, is a useful method for assessing mental stress in adults due to the simplicity of collection [125]. Anxiety, sadness, and those at high risk of schizophrenia can all be identified using salivary cortisol as a biomarker [126].

Salivary lysozyme levels were significantly lower in students’ pre-test samples compared to post-test samples, indicating that this salivary enzyme may serve as an anxiety indicator [127]. Research conducted by Yang et al. [128] found that emergency nurses who experience high levels of job-related stress tend to have lower quantities of salivary lysozyme as opposed to nurses who usually perform duties on non-emergency wards.

A continuous state of stress can also lead to disturbed sleep. Thus, the circadian cycle of melatonin release may be determined by measuring its content in saliva. Salivary melatonin may be useful as a biomarker for the diagnosis of depression, according to Ito et al. [129], who found a substantial correlation between levels of salivary melatonin (both at night and during the day) and depression. Thus, melatonin in saliva has the potential to develop into a valuable biomarker for diagnostic purposes down the road.

One acidic glycoprotein that may be detected in saliva is chromogranin A. Scientific evidence shows that stressful situations, such as public speaking, athletic competition, loud noises, taking and passing an exam, receiving medical treatment, or operating a motor vehicle, cause a quick and targeted rise in CgA in saliva [130,131,132,133]. People with anxiety or depression have not had their salivary chromogranin A levels reported. Tests for CgA in saliva have the distinct benefit of being time-independent, unlike the aforementioned stress indicators in saliva (cortisol, alpha-amylase, melatonin).

Along with the aforementioned stress-markers, studies also indicated a rise in the salivary chaperone Hsp70 [134] and a fall in secretory IgA [135]. Potentially, in the coming years, a plethora of additional stress-indicators and stress-responsive markers will be identified by complete scanning of the genome, transcriptome, and proteome detected in saliva.

### 5.9. Neurological Disorders

Psychiatric, neurodevelopmental, and neurodegenerative disorders are all part of the larger family of illnesses known as neurological disorders. Identifying individuals with diseases at an early stage is essential for prescribing the right treatment [136]. However, there are still many obstacles to overcome when it comes to diagnosing disorders affecting the central nervous system.

This is because there are many delays between the diagnosis and the start of therapy, which reduces the efficacy of the treatment. Lumbar puncture or blood tests are typically used to diagnose neurological disorders. The need for more precise, less intrusive diagnostic methods is driven by the fact that these procedures can cause patients discomfort, suffering, and undesirable side effects, particularly with lumbar punctures [137].

One novel and convenient physiological fluid that may be obtained non-invasively and evaluated with several analytical techniques is saliva [138]. The availability of salivary biomarkers for disorders affecting the neurological system is limited and is currently under investigation, despite the fact that many diseases have validated biomarkers in saliva [139]. Recent research suggests that a sensitive and direct method of screening for Alzheimer’s disease might be found in salivary trehalose, which could be detected using certain cell-based extended gate ion-sensitive field-effect transistor biosensors [140].

As an example, Shi et al. [141] discovered that people with Alzheimer’s disease had higher quantities of phosphorylated proteins in their saliva than healthy persons, suggesting that salivary diagnostics may be useful in diagnosing this neurodegenerative illness.

### 5.10. Forensic Medicine

Because of its abundance of biological information, relative ease of collection (no intrusive procedures required), and relative accessibility, saliva has become a pivotal diagnostic tool in forensic research. In criminal investigations and legal situations, the biofluid known as saliva can provide crucial evidence due to its DNA, proteins, hormones, enzymes, and metabolites [142]. Identification, drug detection, and connecting people to crime scenes are just a few of its many forensic uses.

Genomic information carried by epithelial cells in saliva may be utilized for a very accurate individual identification using methods like the polymerase chain reaction [143]. Saliva is crucial in toxicological investigations, in addition to DNA profiling. It may show whether substances like alcohol or narcotics are present. When it comes to pharmacologically active chemicals, saliva is a reliable indication since, unlike blood, it reflects the free, active forms of drugs [144].

### 5.11. Gastrointestinal Diseases

One non-invasive diagnostic medium for gastrointestinal illnesses that has shown promise is saliva. While a large amount of work has been invested into understanding the symptoms of inflammatory bowel disease (IBD), very little is known about how this condition affects saliva production and its characteristics. Salivary biomarkers as calprotectin, lactoferrin, and cytokines as TNF and IL-6 can be used to track inflammatory bowel disease (IBD), which indicates damage to the intestines and systemic inflammation [145].

Also, multiple metabolite concentrations in saliva have been found to fluctuate in inflammatory bowel disease (IBD) patients [146]. They mostly consist of substances that induce inflammation and oxidative damage.

Saliva contains several components, but two that play a key role in local immunity and host defense are myeloperoxidase (MPO) and immunoglobulin A (IgA). As a protective mechanism, IgA opsonises and agglutinates bacteria, preventing them from adhering to the epithelium of the mucosa lining the gastrointestinal system, which includes the mouth cavity [147,148]. Changes in IgA levels have the potential to increase the risk of erosions and ulcers caused by bacteria invading the mucosa [149]. Although myeloperoxidase is involved in neutrophil microbial destruction, it may potentially exacerbate inflammation-related tissue damage [150]. The healing of intestinal mucosa may be detectable by MPO concentrations, according to certain reports [151].

Salivary myeloperoxidase and IgA levels were shown to be lower in UC patients compared to healthy controls and patients with CD, according to recent research [152], but additional research is needed to confirm the efficacy of non-invasive saliva diagnostics in tracking the progression of biological therapy in inflammatory bowel disease patients, although their findings suggest promise.

A new avenue for studies into health issues associated with obesity has opened up since the secretory function of adipose tissue was found. Both the blood and saliva contain adipokines. Multiple studies have shown that adipokine concentrations in saliva are influenced by obesity [153]. Research has indicated that people who are overweight often experience moderate chronic inflammation in their parotid glands. This inflammation is accompanied by inflammatory mediators released by adipose tissue and acts along the hypothalamic–pituitary–adrenal axis. One possible cause of this inflammation is the reduced activity of the salivary glands [154]. Researchers Goodson et al. have verified that a saliva analysis may effectively detect metabolic abnormalities caused by high body weight in children and adolescents (10–12 years old), particularly in young patients for whom a blood sample could be challenging [155].

Better overall health monitoring in obese individuals would be possible with the establishment of reference values for the proinflammatory cytokines in saliva. Systemic remedies for obese people require more investigation.

### 5.12. Saliva in Chronobiology

Human biofluid and tissue metabolomes have been the subject of a great deal of research on time-of-day variation within the last decade. Endogenous circadian rhythms, which are controlled by internal clocks in the brain and spinal cord, or external diurnal rhythms, which are caused by changes in behavior and the environment and show up as regular 24-h cycles of metabolite concentrations, are the potential causes of this daily fluctuation [156].

Particularly in the prevention of oral diseases and the control of oral infections, saliva plays an important role in managing the oral microbiota and sustaining oral health. The rate of salivary flow and secretion were shown to be associated with circadian cycles in recent investigations. Researchers discovered differential expressions of clock gene mRNAs and clock proteins in the serous acini and duct cells of every major salivary gland. In human individuals, a number of substances exhibited strong rhythmicity, including hormones (melatonin and cortisol), growth factors, enzymes, immunoglobulins, and ions (such as Na+, K+, HCO_3_^−^, and Cl^−^) [157].

For non-invasive circadian rhythm monitoring, saliva is a useful tool in chronobiology. Salivary biomarkers like melatonin and cortisol show clear trends throughout the day; therefore, they may be used to track changes in hormones and physiological functions [158]. Most individuals suffer from some kind of sleep disturbance or poor-quality sleep, which has a negative impact on their health and overall quality of life. A number of studies have linked changes in salivary concentrations of stress hormones and inflammatory cytokines to a variety of sleep problems [159,160].

Circadian rhythms govern the secretion of cytokines, including IL-6, which is known to affect sleep patterns and is seen to rise with lethargy [161]. Adults’ daytime plasma IL-6 levels are elevated when they have either insufficient or disturbed sleep [162]. Blood IL-6 levels have been most consistently used in research establishing a connection between sleep and IL-6, rather than saliva.

Problems with using saliva as a diagnostic fluid have arisen from our incomplete knowledge of the biomolecules found in saliva, how they relate to illness etiology, and how these biomolecules change during the day and night [163]. Examining biological variation in relation to diurnal cycles is essential if saliva is to be used as a non-invasive source of biomarkers suggesting systemic illness. There has been little research on the human salivary microbiome’s circadian oscillations thus far, and what little there is has shown mixed results.

Thoroughly investigating the potential daily changes in the oral microbiota and inflammation caused by the circadian rhythm is necessary to completely ascertain the potential therapeutic value of saliva in the future.

Saliva plays a significant role in modern medicine, serving as a non-invasive and highly informative diagnostic tool. Table 2 highlights its applications across various medical fields, showcasing its potential in disease detection, monitoring, and research.

## 6. Salivary Diagnostic Technology

A very new and exciting area of study, salivary diagnostics makes use of human saliva as a cheap, easily available, and non-invasive diagnostic tool [164]. The sensitivity and specificity of salivary diagnostics have been greatly improved with the incorporation of sophisticated technologies such microfluidics, biosensors, and next-generation sequencing. These advancements pave the way for the identification of illness biomarkers in real-time, which in turn allows for more targeted and expedited treatment strategies.

**Figure 2:**
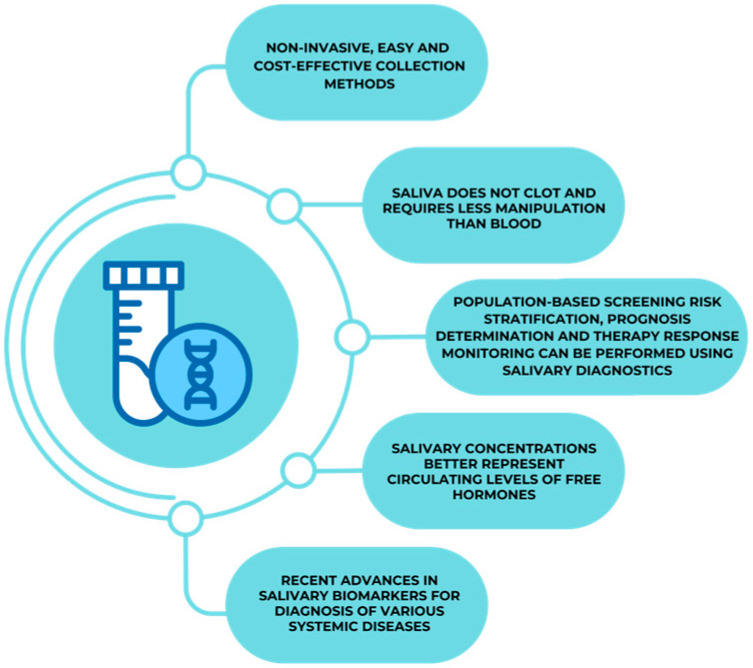
Characteristics that set up saliva as an essential diagnostic tool.

Saliva sampling is safer and more comfortable than traditional blood drawing, which makes it an excellent choice for patients who are young, old, or have impaired immune systems because it is not intrusive [165]. The simplicity of saliva-based methods reduces the need for biohazard disposal compared to blood tests, aligning with sustainable healthcare practices. All these features are captured in Figure 2 below.

## 7. Future Directions

Diagnostic research is heading in a revolutionary new path with the incorporation of wearable technologies for continuous salivary monitoring [166,167]. Conditions like glucose monitoring in diabetics or stress-related hormonal swings might be addressed with quick feedback, made possible by these gadgets that allow for the real-time evaluation of biomarkers. In line with the increasing desire for personalized healthcare, these technologies would increase access to diagnostics [168].

Also, there is a great deal of hope for diagnosing new illnesses and pandemics using saliva. Rapid, non-invasive diagnoses during public health crises may be possible using saliva testing for infections like SARS-CoV-2, which have shown their worth in large-scale screening [110]. To further improve global preparation, more studies into the function of saliva in the early identification of zoonotic infections or antibiotic resistance should be conducted. Ensuring these developments are successful and equitable across various populations will need coordination between technology developers, physicians, and legislators. Only then can we hope to achieve these aims.

## 8. Conclusions

Salivary diagnostics is in the vanguard of contemporary healthcare because of the ever-increasing demand for quick, accurate, and easy-to-use diagnostic tools. A new age of preventative and precision medicine may dawn in this sector, which has tremendous promise for closing affordability and accessibility barriers. Technological progress ensures that salivary diagnostics will continue to grow in usefulness and accuracy, which will have far-reaching consequences for both patients and doctors.

## Figures and Tables

**Table 1:** Salivary biomarkers and their potential applications.

Salivary Biomarkers	Potential Applications
DNA [1]	Standard genotyping Bacterial infection Diagnosing carcinomas of the head and neck Forensics
RNA [2,3]	Viral/bacterial identification carcinomas of the head and neck
Proteins [50]	Diagnosing carcinomas of the head and neck
Mucins/glycoproteins [46]	Diagnosing carcinomas of the head and neck
Immunoglobulins [43]	Diagnosing viruses (HIV, hepatitis B and C)
Drugs and their metabolites [3]	Monitoring drug abuse Detecting drugs in the body
Viruses, bacteria [7,8]	Epstein–Barr virus reactivation (mononucleosis)
Cellular material [65]	Diagnosing carcinomas of the head and neck

**Table 2:** Use of saliva in different medical fields.

Medical Field	Role of Saliva	Applications
Oncology	Diagnostic tool for cancer detection and monitoring	Detection of oral, pancreatic, and gastric cancers through biomarkers like miR-31 and proteins such as CA15-3
Cardiology	Risk assessment for cardiovascular diseases	Use of salivary alpha-amylase and C-reactive protein for detecting myocardial infarctions
Endocrinology	Monitoring hormone levels and endocrine function	Assessment of estradiol, testosterone, and melatonin levels
Diabetes	Biomarker source for glycemic control and disease monitoring	Salivary glucose and α-2-macroglobulin linked to diabetes and HbA1c levels
Neurology	Non-invasive detection of neurodegenerative disorders	Salivary trehalose and phosphorylated proteins for Alzheimer’s disease screening
Infectious Diseases	Identification of pathogens and immune responses	Detection of HIV, hepatitis, and COVID-19 using salivary antibodies and PCR techniques
Forensic Medicine	Source of biological evidence	DNA profiling, drug detection, and toxicological analyses
Gastroenterology	Non-invasive monitoring of gastrointestinal conditions	Use of calprotectin and lactoferrin for tracking inflammatory bowel disease

## References

[1] Pedersen A.M.L., Sørensen C.E., Proctor G.B., Carpenter G.H., Ekström J. (2018). Salivary secretion in health and disease. J. Oral Rehabil..

[2] Woo J.S., Lu D.Y. (2019). Procurement, Transportation, and Storage of Saliva, Buccal Swab, and Oral Wash Specimens. Methods Mol. Biol..

[3] Goto T., Kishimoto T., Iwawaki Y., Fujimoto K., Ishida Y., Watanabe M., Nagao K., Ichikawa T. (2020). Reliability of Screening Methods to Diagnose Oral Dryness and Evaluate Saliva Secretion. Dent. J..

[4] Zhang C.Z., Cheng X.Q., Li J.Y., Zhang P., Yi P., Xu X., Zhou X.D. (2016). Saliva in the diagnosis of diseases. Int. J. Oral Sci..

[5] Dave P.K., Rojas-Cessa R., Dong Z., Umpaichitra V. (2021). Survey of Saliva Components and Virus Sensors for Prevention of COVID-19 and Infectious Diseases. Biosensors.

[6] Hamid H., Khurshid Z., Adanir N., Zafar M.S., Zohaib S. (2020). COVID-19 Pandemic and Role of Human Saliva as a Testing Biofluid in Point-of-Care Technology. Eur. J. Dent..

[7] Roca C., Alkhateeb A.A., Deanhardt B.K., Macdonald J.K., Chi D.L., Wang J.R., Wolfgang M.C. (2024). Saliva sampling method influences oral microbiome composition and taxa distribution associated with oral diseases. PLoS ONE.

[8] McGeachan A.J., Mcdermott C.J. (2017). Management of oral secretions in neurological disease. Pract. Neurol..

[9] Lamy E., Capela-Silva F., Tvarijonaviciute A. (2018). Research on Saliva Secretion and Composition. Biomed. Res. Int..

[10] Helmerhorst E.J., Oppenheim F.G. (2007). Saliva: A dynamic proteome. J. Dent. Res..

[11] Ghods K., Motamed B., Alaee A., Farrokhnia T. (2021). Review of Oral Saliva Measurement Standard Methods. J. Res. Dent. Sci..

[12] Bozorgi C., Holleufer C., Wendin K. (2020). Saliva Secretion and Swallowing—The Impact of Different Types of Food and Drink on Subsequent Intake. Nutrients.

[13] Carpenter G.H. (2013). The secretion, components, and properties of saliva. Annu. Rev. Food Sci. Technol..

[14] Choi S., Shin Y.H., Namkoong E., Hwang S.M., Cong X., Yu G., Park K. (2014). TRPV1 in Salivary Gland Epithelial Cells Is Not Involved in Salivary Secretion via Transcellular Pathway. Korean J. Physiol. Pharmacol..

[15] Suzuki A., Ogata K., Iwata J. (2021). Cell signaling regulation in salivary gland development. Cell. Mol. Life Sci..

[16] Wei F., Wei M.X., Murakami M. (2015). Mechanism involved in Danshen-induced fluid secretion in salivary glands. World J. Gastroenterol..

[17] Kubala E., Strzelecka P., Grzegocka M., Lietz-Kijak D., Gronwald H., Skomro P., Kijak E. (2018). A Review of Selected Studies That Determine the Physical and Chemical Properties of Saliva in the Field of Dental Treatment. Biomed. Res. Int..

[18] Rabiei M., Asli H.N., Mohamadi M.H. (2019). Comparison of Salivary Calcium Level in Dentulous and Edentulous Patients. Eur. J. Dent..

[19] Rajesh K.S., Zareena, Hegde S., Arun Kumar M.S. (2015). Assessment of salivary calcium, phosphate, magnesium, pH, and flow rate in healthy subjects, periodontitis, and dental caries. Contemp. Clin. Dent..

[20] Fiyaz M., Ramesh A., Ramalingam K., Thomas B., Shetty S., Prakash P. (2013). Association of salivary calcium, phosphate, pH and flow rate on oral health: A study on 90 subjects. J. Indian Soc. Periodontol..

[21] Wada M., Orihara K., Kamagata M., Hama K., Sasaki H., Haraguchi A., Miyakawa H., Nakao A., Shibata S. (2017). Circadian clock-dependent increase in salivary IgA secretion modulated by sympathetic receptor activation in mice. Sci. Rep..

[22] Shimazaki Y., Fu B., Yonemoto K., Akifusa S., Shibata Y., Takeshita T., Ninomiya T., Kiyohara Y., Yamashita Y. (2017). Stimulated salivary flow rate and oral health status. J. Oral Sci..

[23] Theda C., Hwang S.H., Czajko A., Loke Y.J., Leong P., Craig J.M. (2018). Quantitation of the cellular content of saliva and buccal swab samples. Sci. Rep..

[24] Budala D.G., Balcos C., Bida F.C., Virvescu D.I., Baciu E.R., Surlari Z. (2021). Changes in saliva secretion in the context of teeth loss. Rom. J. Oral Rehab..

[25] Kanehira T., Hongou H., Asano K., Morita M., Maeshima E., Matsuda A., Sakamoto W. (2014). A simple test for salivary gland function measuring resting and stimulated submandibular and sublingual secretions. Oral Surg. Oral Med. Oral Pathol. Oral Radiol..

[26] Proctor G.B. (2016). The physiology of salivary secretion. Periodontol. 2000.

[27] Wilson K.F., Meier J.D., Ward P.D. (2014). Salivary gland disorders. Am. Fam. Physician.

[28] Swid M.A., Li L., Drahnak E.M., Idom H., Quinones W. (2023). Updated Salivary Gland Immunohistochemistry: A Review. Arch. Pathol. Lab Med..

[29] Hosoi K., Yao C., Hasegawa T., Yoshimura H., Akamatsu T. (2020). Dynamics of Salivary Gland AQP5 under Normal and Pathologic Conditions. Int. J. Mol. Sci..

[30] Tanasiewicz M., Hildebrandt T., Obersztyn I. (2016). Xerostomia of Various Etiologies: A Review of the Literature. Adv. Clin. Exp. Med..

[31] Porcheri C., Mitsiadis T.A. (2019). Physiology, Pathology and Regeneration of Salivary Glands. Cells.

[32] Tan E.C.K., Lexomboon D., Sandborgh-Englund G., Haasum Y. (2018). Medications That Cause Dry Mouth as an Adverse Effect in Older People: A Systematic Review and Metaanalysis. J. Am. Geriatr. Soc..

[33] López-Jornet P., Collado Y., Zambudio A., Pons-Fuster E., Castillo Felipe C., Tvarijonaviciute A. (2021). Chemosensory Function in Burning Mouth Syndrome a Comparative Cross-Sectional Study. Nutrients.

[34] Toan N., Ahn S.-G. (2021). Aging-Related Metabolic Dysfunction in the Salivary Gland: A Review of the Literature. Int. J. Mol. Sci..

[35] Kapourani A., Kontogiannopoulos K.N., Manioudaki A.-E., Poulopoulos A.K., Tsalikis L., Assimopoulou A.N., Barmpalexis P. (2022). A Review on Xerostomia and Its Various Management Strategies: The Role of Advanced Polymeric Materials in the Treatment Approaches. Polymers.

[36] Mortazavi H., Baharvand M., Movahhedian A., Mohammadi M., Khodadoustan A. (2014). Xerostomia due to systemic disease: A review of 20 conditions and mechanisms. Ann. Med. Health Sci. Res..

[37] Ugga L., Ravanelli M., Pallottino A.A., Farina D., Maroldi R. (2017). Diagnostic work-up in obstructive and inflammatory salivary gland disorders. Acta Otorhinolaryngol. Ital..

[38] Thomson W.M., Smith M.B., Ferguson C.A., Moses G. (2021). The Challenge of Medication-Induced Dry Mouth in Residential Aged Care. Pharmacy.

[39] Villa A., Abati S. (2011). Risk factors and symptoms associated with xerostomia: A cross-sectional study. Aust. Dent. J..

[40] Hast M.A., Kong A.M., Abdelhadi J., Shah R., Szendrey A., Holmes J. (2024). Real-World Observational Analysis of Clinical Characteristics and Treatment Patterns of Patients with Chronic Sialorrhea. Toxins.

[41] Jost W.H., Bäumer T., Laskawi R., Slawek J., Spittau B., Steffen A., Winterholler M., Bavikatte G. (2019). Therapy of Sialorrhea with Botulinum Neurotoxin. Neurol. Ther..

[42] Wolff A., Joshi R.K., Ekström J., Aframian D., Pedersen A.M., Proctor G., Narayana N., Villa A., Sia Y.W., Aliko A. (2017). A Guide to Medications Inducing Salivary Gland Dysfunction, Xerostomia, and Subjective Sialorrhea: A Systematic Review Sponsored by the World Workshop on Oral Medicine VI. Drugs R D.

[43] Vila T., Rizk A.M., Sultan A.S., Jabra-Rizk M.A. (2019). The power of saliva: Antimicrobial and beyond. PLoS Pathog..

[44] Wertz P.W., de Szalay S. (2020). Innate Antimicrobial Defense of Skin and Oral Mucosa. Antibiotics.

[45] Matczuk J., Żendzian-Piotrowska M., Maciejczyk M., Kurek K. (2017). Salivary lipids: A review. Adv. Clin. Exp. Med..

[46] Frenkel E.S., Ribbeck K. (2015). Salivary mucins in host defense and disease prevention. J. Oral Microbiol..

[47] Uchida H., Ovitt C.E. (2022). Novel impacts of saliva with regard to oral health. J. Prosthet. Dent..

[48] Tiwari M. (2011). Science behind human saliva. J. Nat. Sci. Biol. Med..

[49] Song M., Bai H., Zhang P., Zhou X., Ying B. (2023). Promising applications of human-derived saliva biomarker testing in clinical diagnostics. Int. J. Oral Sci..

[50] Kraaij S., de Visscher J.G.A.M., Apperloo R.C., Nazmi K., Bikker F.J., Brand H.S. (2023). Lactoferrin and the development of salivary stones: A pilot study. Biometals.

[51] Wang J., Liang Y., Wang Y., Cui J., Liu M., Du W., Xu Y. (2013). Computational prediction of human salivary proteins from blood circulation and application to diagnostic biomarker identification. PLoS ONE.

[52] Fábián T.K., Hermann P., Beck A., Fejérdy P., Fábián G. (2012). Salivary Defense Proteins: Their Network and Role in Innate and Acquired Oral Immunity. Int. J. Mol. Sci..

[53] Li Q., Ouyang X., Chen J., Zhang P., Feng Y. (2020). A Review on Salivary Proteomics for Oral Cancer Screening. Curr. Issues Mol. Biol..

[54] Chibly A.M., Aure M.H., Patel V.N., Hoffman M.P. (2022). Salivary gland function, development, and regeneration. Physiol. Rev..

[55] Kroese F.G.M., Haacke E.A., Bombardieri M. (2018). The role of salivary gland histopathology in primary Sjögren’s syndrome: Promises and pitfalls. Clin. Exp. Rheumatol..

[56] Nonaka T., Wong D.T.W. (2022). Saliva Diagnostics. Annu. Rev. Anal. Chem..

[57] Martin L.E., Gutierrez V.A., Torregrossa A.M. (2023). The role of saliva in taste and food intake. Physiol. Behav..

[58] Feng Y., Licandro H., Martin C., Septier C., Zhao M., Neyraud E., Morzel M. (2018). The Associations between Biochemical and Microbiological Variables and Taste Differ in Whole Saliva and in the Film Lining the Tongue. Biomed. Res. Int..

[59] López-Dávalos P.C., Requena T., Pozo-Bayón M.Á., Muñoz-González C. (2023). Decreased retronasal olfaction and taste perception in obesity are related to saliva biochemical and microbiota composition. Food. Res. Int..

[60] Błochowiak K. (2022). Smell and Taste Function and Their Disturbances in Sjögren’s Syndrome. Int. J. Environ. Res. Public Health.

[61] Fábián T.K., Beck A., Fejérdy P., Hermann P., Fábián G. (2015). Molecular Mechanisms of Taste Recognition: Considerations about the Role of Saliva. Int. J. Mol. Sci..

[62] Sivakumar A., Narayanan R. (2024). Comparison of Salivary Flow Rate, pH, Buffering Capacity, and Secretory Immunoglobulin A Levels between Children with Early Childhood Caries and Caries-free Children. Int. J. Clin. Pediatr. Dent..

[63] Madariaga V.I., Pereira-Cenci T., Walboomers X.F., Loomans B.A.C. (2023). Association between salivary characteristics and tooth wear: A systematic review and meta-analysis. J. Dent..

[64] Andrioaie I.M., Luchian I., Dămian C., Nichitean G., Andrese E.P., Pantilimonescu T.F., Trandabăț B., Prisacariu L.J., Budală D.G., Dimitriu D.C. (2023). The Clinical Utility of Circulating HPV DNA Biomarker in Oropharyngeal, Cervical, Anal, and Skin HPV-Related Cancers: A Review. Pathogens.

[65] Schweigel H., Wicht M., Schwendicke F. (2016). Salivary and pellicle proteome: A datamining analysis. Sci. Rep..

[66] Miller C.S., Foley J.D., Bailey A.L., Campell C.L., Humphries R.L., Christodoulides N., Floriano P.N., Simmons G., Bhagwandin B., Jacobson J.W. (2010). Current developments in salivary diagnostics. Biomark. Med..

[67] Caselli E., Fabbri C., D’Accolti M., Soffritti I., Bassi C., Mazzacane S., Franchi M. (2020). Defining the oral microbiome by whole-genome sequencing and resistome analysis: The complexity of the healthy picture. BMC Microbiol..

[68] Roi A., Rusu L.C., Roi C.I., Luca R.E., Boia S., Munteanu R.I. (2019). A New Approach for the Diagnosis of Systemic and Oral Diseases Based on Salivary Biomolecules. Dis. Markers.

[69] Siegel R., Naishadham D., Jemal A. (2013). Cancer statistics, 2013. CA Cancer J. Clin..

[70] Humeau M., Vignolle-Vidoni A., Sicard F., Martins F., Bournet B., Buscail L., Torrisani J., Cordelier P. (2015). Salivary MicroRNA in pancreatic cancer patients. PLoS ONE.

[71] Wei A.L., Li M., Li G.Q., Wang X., Hu W.M., Li Z.L., Yuan J., Liu H.Y., Zhou L.L., Li K. (2020). Oral microbiome and pancreatic cancer. World J. Gastroenterol..

[72] Yang W.J., Zhao H.P., Yu Y., Wang J.H., Guo L., Liu J.Y., Pu J., Lv J. (2023). Updates on global epidemiology, risk and prognostic factors of gastric cancer. World J. Gastroenterol..

[73] Maconi G., Manes G., Porro G.B. (2008). Role of symptoms in diagnosis and outcome of gastric cancer. World J. Gastroenterol..

[74] Xiao H., Zhang Y., Kim Y., Kim S., Kim J.J., Kim K.M., Yoshizawa J., Fan L.Y., Cao C.X., Wong D.T. (2016). Differential proteomic analysis of human saliva using tandem mass tags quantification for gastric cancer detection. Sci. Rep..

[75] Laidi F., Bouziane A., Lakhdar A., Khabouze S., Amrani M., Rhrab B., Zaoui F. (2014). Significant correlation between salivary and serum Ca 15-3 in healthy women and breast cancer patients. Asian Pac. J. Cancer Prev..

[76] Wood N., Streckfus C.F. (2015). The Expression of Lung Resistance Protein in Saliva: A Novel Prognostic Indicator Protein for Carcinoma of the Breast. Cancer Investig..

[77] Arantes L.M., de Carvalho A.C., Melendez M.E., Carvalho A.L., Goloni-Bertollo E.M. (2014). Methylation as a biomarker for head and neck cancer. Oral Oncol..

[78] Dhanuthai K., Rojanawatsirivej S., Thosaporn W., Kintarak S., Subarnbhesaj A., Darling M., Kryshtalskyj E., Chiang C.P., Shin H.I., Choi S.Y. (2018). Oral cancer: A multicenter study. Med. Oral Patol. Oral Cir. Bucal..

[79] Ovchinnikov D.A., Cooper M.A., Pandit P., Coman W.B., Cooper-White J.J., Keith P., Wolvetang E.J., Slowey P.D., Punyadeera C. (2012). Tumor-suppressor Gene Promoter Hypermethylation in Saliva of Head and Neck Cancer Patients. Transl. Oncol..

[80] Park N.J., Zhou H., Elashoff D., Henson B.S., Kastratovic D.A., Abemayor E., Wong D.T. (2009). Salivary microRNA: Discovery, characterization, and clinical utility for oral cancer detection. Clin. Cancer Res..

[81] Liu C.J., Lin S.C., Yang C.C., Cheng H.W., Chang K.W. (2012). Exploiting salivary miR-31 as a clinical biomarker of oral squamous cell carcinoma. Head Neck..

[82] Duz M.B., Karatas O.F., Guzel E., Turgut N.F., Yilmaz M., Creighton C.J., Ozen M. (2016). Identification of miR-139-5p as a saliva biomarker for tongue squamous cell carcinoma: A pilot study. Cell. Oncol..

[83] Wong D.T. (2012). Salivary diagnostics. Oper. Dent..

[84] Aziz S., Ahmed S.S., Ali A., Khan F.A., Zulfiqar G., Iqbal J., Khan A.A., Shoaib M. (2015). Salivary Immunosuppressive Cytokines IL-10 and IL-13 Are Significantly Elevated in Oral Squamous Cell Carcinoma Patients. Cancer Invest..

[85] Allegra E., Trapasso S., La Boria A., Aragona T., Pisani D., Belfiore A., Garozzo A. (2014). Prognostic role of salivary CD44sol levels in the follow-up of laryngeal carcinomas. J. Oral Pathol. Med..

[86] Yuvaraj M., Udayakumar K., Jayanth V., Prakasa Rao A., Bharanidharan G., Koteeswaran D., Munusamy B.D., Murali Krishna C., Ganesan S. (2014). Fluorescence spectroscopic characterization of salivary metabolites of oral cancer patients. J. Photochem. Photobiol. B..

[87] Tantray S., Sharma S., Prabhat K., Nasrullah N., Gupta M. (2022). Salivary metabolite signatures of oral cancer and leukoplakia through gas chromatography-mass spectrometry. J. Oral Maxillofac. Pathol..

[88] Corcoran R.B., Chabner B.A. (2018). Application of Cell-free DNA Analysis to Cancer Treatment. N. Engl. J. Med..

[89] Guo Z.W., Xiao W.W., Yang X.X., Yang X., Cai G.X., Wang X.J., Han B.W., Li K., Zhai X.M., Li F.X. (2020). Noninvasive prediction of response to cancer therapy using promoter profiling of circulating cell-free DNA. Clin. Transl. Med..

[90] Spindler K.L., Pallisgaard N., Vogelius I., Jakobsen A. (2012). Quantitative cell-free DNA, KRAS, and BRAF mutations in plasma from patients with metastatic colorectal cancer during treatment with cetuximab and irinotecan. Clin. Cancer Res..

[91] Diaz L.A., Williams R.T., Wu J., Kinde I., Hecht J.R., Berlin J., Allen B., Bozic I., Reiter J.G., Nowak M.A. (2012). The molecular evolution of acquired resistance to targeted EGFR blockade in colorectal cancers. Nature.

[92] Ozbay Y., Aydin S., Dagli A.F., Akbulut M., Dagli N., Kilic N., Rahman A., Sahin I., Polat V., Ozercan H.I. (2008). Obestatin is present in saliva: Alterations in obestatin and ghrelin levels of saliva and serum in ischemic heart disease. BMB Rep..

[93] Miller C.S., Foley J.D., Floriano P.N., Christodoulides N., Ebersole J.L., Campbell C.L., Bailey A.L., Rose B.G., Kinane D.F., Novak M.J. (2014). Utility of salivary biomarkers for demonstrating acute myocardial infarction. J. Dent. Res..

[94] Shen Y.S., Chen W.L., Chang H.Y., Kuo H.Y., Chang Y.C., Chu H. (2012). Diagnostic performance of initial salivary alpha-amylase activity for acute myocardial infarction in patients with acute chest pain. J. Emerg. Med..

[95] Floriano P.N., Christodoulides N., Millerm C.S., Ebersole J.L., Spertus J., Rose B.G., Kinane D.F., Novak M.J., Steinhubl S., Acosta S. (2009). Use of saliva-based nano-biochip tests for acute myocardial infarction at the point of care: A feasibility study. Clin. Chem..

[96] Zheng H., Li R., Zhang J., Zhou S., Ma Q., Zhou Y., Chen F., Lin J. (2014). Salivary biomarkers indicate obstructive sleep apnea patients with cardiovascular diseases. Sci. Rep..

[97] Giannoni M., Consales V., Campanati A., Ganzetti G., Giuliodori K., Postacchini V., Liberati G., Azzaretto L., Vichi S., Guanciarossa F. (2015). Homocysteine plasma levels in psoriasis patients: Our experience and review of the literature. J. Eur. Acad. Dermatol. Venereol..

[98] Luchetti M.M., Benfaremo D., Campanati A., Molinelli E., Ciferri M., Cataldi S., Capeci W., Di Carlo M., Offidani A.M., Salaffi F. (2018). Clinical outcomes and feasibility of the multidisciplinary management of patients with psoriatic arthritis: Two-year clinical experience of a dermo-rheumatologic clinic. Clin. Rheumatol..

[99] Asa’ad F., Fiore M., Alfieri A., Pigatto P.D.M., Franchi C., Berti E., Maiorana C., Damiani G. (2018). Saliva as a Future Field in Psoriasis Research. BioMed. Res. Int..

[100] Soudan R.A., Daoud S.A., Mashlah A.M. (2011). Study of some salivary changes in cutaneous psoriatic patients. Saudi Med. J..

[101] Belstrøm D., Eiberg J.M., Enevold C., Grande M.A., Jensen C.A.J., Skov L., Hansen P.R. (2020). Salivary microbiota and inflammation-related proteins in patients with psoriasis. Oral Dis..

[102] Luchian I., Martu I., Ioanid N., Goriuc A., Vata I., Martu Stefanache A., Hurjui L., Tatarciuc M., Matei M.N., Martu S. (2016). Salivary interleukin-1β: A biochemical marker that predicts periodontal disease in orthodontic treatment. Rev. Chim..

[103] Ganzetti G., Campanati A., Santarelli A., Pozzi V., Molinelli E., Minnetti I., Brisigotti V., Procaccini M., Emanuelli M., Offidani A. (2015). Involvement of the oral cavity in psoriasis: Results of a clinical study. Br. J. Dermatol..

[104] Andreadis D., Lorenzini G., Drakoulakos D., Belazi M., Mihailidou E., Velkos G., Mourellou-Tsatsou O., Antoniades D. (2006). Detection of pemphigus desmoglein 1 and desmoglein 3 autoantibodies and pemphigoid BP180 autoantibodies in saliva and comparison with serum values. Eur. J. Oral Sci..

[105] Campanati A., Martina E., Diotallevi F., Radi G., Marani A., Sartini D., Emanuelli M., Kontochristopoulos G., Rigopoulos D., Gregoriou S. (2021). Saliva Proteomics as Fluid Signature of Inflammatory and Immune-Mediated Skin Diseases. Int. J. Mol. Sci..

[106] Fang Y., Xie H., Fan C. (2022). Association of hypertension with *Helicobacter pylori*: A systematic review and meta-analysis. PLoS ONE.

[107] Gisbert J.P. (2015). *Helicobacter pylori*-associated diseases. Gastroenterol. Hepatol..

[108] Young S.H., Luo J.C. (2016). Will saliva test be a good method to detect *Helicobacter pylori* in *H. pylori*-infected patients?. J. Chin. Med. Assoc..

[109] Corstjens P.L., Abrams W.R., Malamud D. (2016). Saliva and viral infections. Periodontol 2000.

[110] Oliveira Neto N.F.D., Caixeta R.A.V., Zerbinati R.M., Zarpellon A.C., Caetano M.W., Pallos D., Junges R., Costa A.L.F., Aitken-Saavedra J., Giannecchini S. (2024). The Emergence of Saliva as a Diagnostic and Prognostic Tool for Viral Infections. Viruses.

[111] Nefzi F., Ben Salem N.A., Khelif A., Feki S., Aouni M., Gautheret-Dejean A. (2015). Quantitative analysis of human herpesvirus-6 and human cytomegalovirus in blood and saliva from patients with acute leukemia. J. Med. Virol..

[112] Sheikhakbari S., Mokhtari-Azad T., Salimi V., Norouzbabaei Z., Abbasi S., Zahraei S.M., Shahmahmoodi S. (2012). The use of oral fluid samples spotted on filter paper for the detection of measles virus using nested rt-PCR. J. Clin. Lab. Anal..

[113] Laxton C.S., Peno C., Hahn A.M., Allicock O.M., Perniciaro S., Wyllie A.L. (2023). The potential of saliva as an accessible and sensitive sample type for the detection of respiratory pathogens and host immunity. Lancet Microbe.

[114] Tobik E.R., Kitfield-Vernon L.B., Thomas R.J., Steel S.A., Tan S.H., Allicock O.M., Choate B.L., Akbarzada S., Wyllie A.L. (2022). Saliva as a sample type for SARS-CoV-2 detection: Implementation successes and opportunities around the globe. Expert. Rev. Mol. Diagn..

[115] Sueki A., Matsuda K., Yamaguchi A., Uehara M., Sugano M., Uehara T., Honda T. (2016). Evaluation of saliva as diagnostic materials for influenza virus infection by PCR-based assays. Clin. Chim. Acta.

[116] Kim Y.G., Yun S.G., Kim M.Y., Park K., Cho C.H., Yoon S.Y., Nam M.H., Lee C.K., Cho Y.J., Lim C.S. (2016). Comparison between Saliva and Nasopharyngeal Swab Specimens for Detection of Respiratory Viruses by Multiplex Reverse Transcription-PCR. J. Clin. Microbiol..

[117] Aitken J.P., Ortiz C., Morales-Bozo I., Rojas-Alcayaga G., Baeza M., Beltran C., Escobar A. (2015). α-2-macroglobulin in saliva is associated with glycemic control in patients with type 2 diabetes mellitus. Dis. Markers.

[118] Goriuc A., Cojocaru K.-A., Luchian I., Ursu R.-G., Butnaru O., Foia L. (2024). Using 8-Hydroxy-2′-Deoxiguanosine (8-OHdG) as a Reliable Biomarker for Assessing Periodontal Disease Associated with Diabetes. Int. J. Mol. Sci..

[119] Barnes V.M., Kennedy A.D., Panagakos F., Devizio W., Trivedi H.M., Jönsson T., Guo L., Cervi S., Scannapieco F.A. (2014). Global metabolomic analysis of human saliva and plasma from healthy and diabetic subjects, with and without periodontal disease. PLoS ONE.

[120] Cenzato N., Cazzaniga F., Maspero C., Tartaglia G.M., Del Fabbro M. (2023). SALIVA-based diagnostic approach for diabetes mellitus: A step towards non-invasive detection—A scoping review. Eur. Rev. Med. Pharmacol. Sci..

[121] Aitken-Saavedra J., Rojas-Alcayaga G., Maturana-Ramirez A., Escobar-Alvarez A., Cortes-Coloma A., Reyes-Rojas M., Viera-Sapiain V., Villablanca-Martínez C., Morales-Bozo I. (2015). Salivary gland dysfunction markers in type 2 diabetes mellitus patients. J. Clin. Exp. Dent..

[122] Rzepka-Migut B., Paprocka J. (2020). Melatonin-Measurement Methods and the Factors Modifying the Results. A Systematic Review of the Literature. Int. J. Environ. Res. Public Health.

[123] Amatoury M., Lee J.W., Maguire A.M., Ambler G.R., Steinbeck K.S. (2016). Utility of salivary enzyme immunoassays for measuring estradiol and testosterone in adolescents: A pilot study. Int. J. Adolesc. Med. Health.

[124] Venkatapathy R., Govindarajan V., Oza N., Parameswaran S., Pennagaram Dhanasekaran B., Prashad K.V. (2014). Salivary creatinine estimation as an alternative to serum creatinine in chronic kidney disease patients. Int. J. Nephrol..

[125] Giacomello G., Scholten A., Parr M.K. (2020). Current methods for stress marker detection in saliva. J. Pharm. Biomed. Anal..

[126] Coulon N., Brailly-Tabard S., Walter M., Tordjman S. (2016). Altered circadian patterns of salivary cortisol in individuals with schizophrenia: A critical literature review. J. Physiol. Paris.

[127] Muraoka M.Y., Justino A.B., Caixeta D.C., Queiroz J.S., Sabino-Silva R., Salmen Espindola F. (2022). Fructose and methylglyoxal-induced glycation alters structural and functional properties of salivary proteins, albumin and lysozyme. PLoS ONE.

[128] Agha N.H., Baker F.L., Kunz H.E., Spielmann G., Mylabathula P.L., Rooney B.V., Mehta S.K., Pierson D.L., Laughlin M.S., Markofski M.M. (2020). Salivary antimicrobial proteins and stress biomarkers are elevated during a 6-month mission to the International Space Station. J. Appl. Physiol. 1985.

[129] Ito Y., Iida T., Yamamura Y., Teramura M., Nakagami Y., Kawai K., Nagamura Y., Teradaira R. (2013). Relationships between Salivary Melatonin Levels, Quality of Sleep, and Stress in Young Japanese Females. Int. J. Tryptophan Res..

[130] Vora K.M., Shah P.P., Patil K.V., Kunte S.S., Jagtap C.M., Davalbhakta R.N. (2024). Quantification of Salivary Chromogranin A Levels during Routine Dental Procedures in Children: An In Vivo Study. Int. J. Clin. Pediatr. Dent..

[131] Tammayan M., Jantaratnotai N., Pachimsawat P. (2021). Differential responses of salivary cortisol, amylase, and chromogranin A to academic stress. PLoS ONE.

[132] Dia M.M., Bocanegra O.L., Teixeira R.R., Soares S.S., Espindola F.S. (2012). Response of salivary markers of autonomicactivity to elite competition. Int. J. Sports Med..

[133] Takatsuji K., Sugimoto Y., Ishizaki S., Ozaki Y., Matsuyama E., Yamaguchi Y. (2008). The effects of examination stresson salivary cortisol, immunoglobulin A, and chromogranin A in nursing students. Biomed. Res..

[134] Motahari P., Pourzare Mehrbani S., Jabbarvand H. (2021). Evaluation of Salivary Level of Heat Shock Protein 70 in Patients with Chronic Periodontitis. J. Dent..

[135] Chojnowska S., Ptaszyńska-Sarosiek I., Kępka A., Knaś M., Waszkiewicz N. (2021). Salivary Biomarkers of Stress, Anxiety and Depression. J. Clin. Med..

[136] Manera V., Rovini E., Wais P. (2023). Editorial: Early detection of neurodegenerative disorders using behavioral markers and new technologies: New methods and perspectives. Front. Aging Neurosci..

[137] Kim K.T. (2022). Lumbar puncture: Considerations, procedure, and complications. Encephalitis.

[138] Khanagar S.B., Al-Ehaideb A., Maganur P.C., Vishwanathaiah S., Patil S., Baeshen H.A., Sarode S.C., Bhandi S. (2021). Developments, application, and performance of artificial intelligence in dentistry—A systematic review. J. Dent. Sci..

[139] Boroumand M., Olianas A., Cabras T., Manconi B., Fanni D., Faa G., Desiderio C., Messana I., Castagnola M. (2021). Saliva, a bodily fluid with recognized and potential diagnostic applications. J. Sep. Sci..

[140] Lau H.C., Lee I.K., Ko P.W., Lee H.W., Huh J.S., Cho W.J., Lim J.O. (2015). Non-invasive screening for Alzheimer’s disease by sensing salivary sugar using Drosophila cells expressing gustatory receptor (Gr5a) immobilized on an extended gate ion-sensitive field-effect transistor (EG-ISFET) biosensor. PLoS ONE.

[141] Shi M., Sui Y.T., Peskind E.R., Li G., Hwang H., Devic I., Ginghina C., Edgar J.S., Pan C., Goodlett D.R. (2011). Salivary tau species are potential biomarkers of Alzheimer’s disease. J. Alzheimers Dis..

[142] Kayser M., Branicki W., Parson W., Phillips C. (2023). Recent advances in Forensic DNA Phenotyping of appearance, ancestry and age. Forensic Sci. Int. Genet..

[143] Neis M., Groß T., Schneider H., Schneider P.M., Courts C. (2024). Comprehensive body fluid identification and contributor assignment by combining targeted sequencing of mRNA and coding region SNPs. Forensic Sci. Int. Genet..

[144] Upadhyay M., Shrivastava P., Verma K., Joshi B. (2023). Recent advancements in identification and detection of saliva as forensic evidence: A review. Egypt J. Forensic Sci..

[145] Nijakowski K., Surdacka A. (2020). Salivary Biomarkers for Diagnosis of Inflammatory Bowel Diseases: A Systematic Review. Int. J. Mol. Sci..

[146] Nijakowski K., Rutkowski R., Eder P., Korybalska K., Witowski J., Surdacka A. (2021). Changes in Salivary Parameters of Oral Immunity after Biologic Therapy for Inflammatory Bowel Disease. Life.

[147] Räisänen L., Agrawal N., Mathew B., Kääriäinen S., Kolho K.-L., Viljakainen H. (2023). Pre-Diagnostic Saliva Microbiota of School-Aged Children Who Developed Type 1 Diabetes or Inflammatory Bowel Diseases. Int. J. Mol. Sci..

[148] Kang S.-B., Kim H., Kim S., Kim J., Park S.-K., Lee C.-W., Kim K.O., Seo G.-S., Kim M.S., Cha J.M. (2023). Potential Oral Microbial Markers for Differential Diagnosis of Crohn’s Disease and Ulcerative Colitis Using Machine Learning Models. Microorganisms.

[149] Gürsoy M., Rautava J., Pussinen P., Kristoffersen A.K., Enersen M., Loimaranta V., Gürsoy U.K. (2023). Salivary IgA and IgG Antibody Responses against Periodontitis-Associated Bacteria in Crohn’s Disease. Int. J. Mol. Sci..

[150] Nijakowski K., Motylewska B., Banasik E., Rutkowski R., Tsaryk V., Łuczak J., Korybalska K., Witowski J., Surdacka A., Eder P. (2024). Treatment regimens and disease activity could alter salivary myeloperoxidase levels in patients with inflammatory bowel diseases. Pol. Arch Intern. Med..

[151] Nijakowski K., Jankowski J., Gruszczyński D., Surdacka A. (2023). Salivary Alterations of Myeloperoxidase in Patients with Systemic Diseases: A Systematic Review. Int. J. Mol. Sci..

[152] Nijakowski K., Rutkowski R., Eder P., Simon M., Korybalska K., Witowski J., Surdacka A. (2021). Potential Salivary Markers for Differential Diagnosis of Crohn’s Disease and Ulcerative Colitis. Life.

[153] Lehmann A.P., Nijakowski K., Swora-Cwynar E., Łuczak J., Czepulis N., Surdacka A. (2020). Characteristics of salivary inflammation in obesity. Pol. Arch Intern. Med..

[154] Amar S., Zhou Q., Shaik-Dasthagirisaheb Y., Leeman S. (2007). Diet-induced obesity in mice causes changes in immune responses and bone loss manifested by bacterial challenge. Proc. Natl. Acad. Sci. USA.

[155] Goodson J.M., Kantarci A., Hartman M.L., Denis G.V., Stephens D., Hasturk H., Yaskell T., Vargas J., Wang X., Cugini M. (2014). Metabolic disease risk in children by salivary biomarker analysis. PLoS ONE.

[156] Hancox T.P.M., Skene D.J., Dallmann R., Dunn W.B. (2021). Tick-Tock Consider the Clock: The Influence of Circadian and External Cycles on Time of Day Variation in the Human Metabolome—A Review. Metabolites.

[157] Feng G., Zhao J., Peng J., Luo B., Zhang J., Chen L., Xu Z. (2022). Circadian clock-A promising scientific target in oral science. Front. Physiol..

[158] Yennurajalingam S., Kang D.-H., Hwu W.-J., Padhye N.S., Masino C., Dibaj S.S., Liu D.D., Williams J.L., Lu Z., Bruera E. (2018). Cranial Electrotherapy Stimulation for the Management of Depression, Anxiety, Sleep Disturbance, and Pain in Patients with Advanced Cancer: A Preliminary Study. J. Pain Symptom Manag..

[159] Boström A., Scheele D., Stoffel-Wagner B., Hönig F., Chaudhry S.R., Muhammad S., Hurlemann R., Krauss J.K., Lendvai I.S., Chakravarthy K. (2019). Saliva molecular inflammatory profiling in female migraine patients responsive to adjunctive cervical non-invasive vagus nerve stimulation: The MOXY study. J. Transl. Med..

[160] LaVoy E.C., Palmer C.A., So C., Alfano C.A. (2020). Bidirectional relationships between sleep and biomarkers of stress and immunity in youth. Int. J. Psychophysiol..

[161] Rose-John S. (2018). Interleukin-6 Family Cytokines. Cold Spring Harb. Perspect. Biol..

[162] Sarkar A., Kuehl M.N., Alman A.C., Burkhardt B.R. (2021). Linking the oral microbiome and salivary cytokine abundance to circadian oscillations. Sci. Rep..

[163] Chai-Coetzer C.L., Antic N.A., Rowland L.S., Catcheside P.G., Esterman A., Reed R.L., Williams H., Dunn S.V., McEvoy R.D. (2011). A simplified model of screening questionnaire and home monitoring for obstructive sleep apnoea in primary care. Thorax.

[164] Surdu A., Budala D.G., Luchian I., Foia L.G., Botnariu G.E., Scutariu M.M. (2024). Using AI in Optimizing Oral and Dental Diagnoses—A Narrative Review. Diagnostics.

[165] Abraham J.E., Maranian M.J., Spiteri I., Russell R., Ingle S., Luccarini C., Earl H.M., Pharoah P.P., Dunning A.M., Caldas C. (2012). Saliva samples are a viable alternative to blood samples as a source of DNA for high throughput genotyping. BMC Med. Genom..

[166] Ichikawa K., Iitani K., Kawase G., Toma K., Arakawa T., Dao D.V., Mitsubayashi K. (2024). Mouthguard-Type Wearable Sensor for Monitoring Salivary Turbidity to Assess Oral Hygiene. Sensors.

[167] Surlari Z., Budală D.G., Lupu C.I., Stelea C.G., Butnaru O.M., Luchian I. (2023). Current Progress and Challenges of Using Artificial Intelligence in Clinical Dentistry—A Narrative Review. J. Clin. Med..

[168] Puchakayala S., Umamahesh B., Shien-Ping F. (2022). Wireless accessing of salivary biomarkers based wearable electrochemical sensors: A mini-review. Electrochem. Commun..

